# A Groupwise Association Test for Rare Mutations Using a Weighted Sum Statistic

**DOI:** 10.1371/journal.pgen.1000384

**Published:** 2009-02-13

**Authors:** Bo Eskerod Madsen, Sharon R. Browning

**Affiliations:** 1 Bioinformatics Research Center (BiRC), University of Aarhus, Aarhus C, Denmark; 2 Department of Statistics, The University of Auckland, Auckland, New Zealand; University of California San Diego and The Scripps Research Institute, United States of America

## Abstract

Resequencing is an emerging tool for identification of rare disease-associated mutations. Rare mutations are difficult to tag with SNP genotyping, as genotyping studies are designed to detect common variants. However, studies have shown that genetic heterogeneity is a probable scenario for common diseases, in which multiple rare mutations together explain a large proportion of the genetic basis for the disease. Thus, we propose a weighted-sum method to jointly analyse a group of mutations in order to test for groupwise association with disease status. For example, such a group of mutations may result from resequencing a gene. We compare the proposed weighted-sum method to alternative methods and show that it is powerful for identifying disease-associated genes, both on simulated and Encode data. Using the weighted-sum method, a resequencing study can identify a disease-associated gene with an overall population attributable risk (PAR) of 2%, even when each individual mutation has much lower PAR, using 1,000 to 7,000 affected and unaffected individuals, depending on the underlying genetic model. This study thus demonstrates that resequencing studies can identify important genetic associations, provided that specialised analysis methods, such as the weighted-sum method, are used.

## Author Summary

Resequencing is an emerging tool for the identification of rare disease-associated mutations. Recent studies have shown that groups of multiple rare mutations together can explain a large proportion of the genetic basis for some diseases. Therefore, we propose a new statistical method for analysing a group of mutations in order to test for groupwise association with disease status. We compare the proposed weighted-sum method to alternative methods and show that it is powerful for identifying disease-associated groups of mutations, both on computer-simulated and real data. By using computer simulations, we further show that resequencing a few thousand individuals is sufficient to perform a genome-wide study of all human genes, if the proposed method is used. This study thus demonstrates that resequencing studies can identify important genetic associations, provided that specialised analysis methods, such as the proposed weighted-sum method, are used.

## Introduction

**Figure 1:**
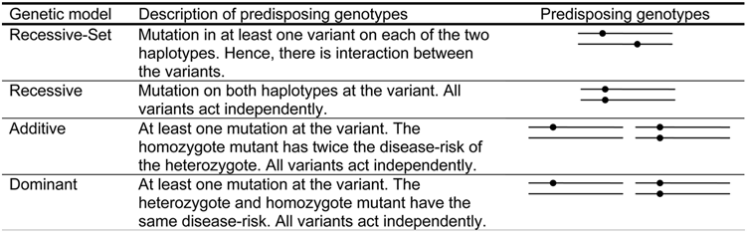
Genetic models. Model descriptions and examples of predisposing genotypes are shown for the genetic models used. Lines symbolise haplotypes and dots symbolise disease-risk mutations.

New technologies allow sequencing of parts of the genome of large groups of individuals [1], and hereby initiate the next generation of large scale association studies. Resequencing studies can directly identify millions of rare mutations in the genome, and may therefore be able to identify disease-mutations that are not tagged by panels of common SNPs [2]. Resequencing may thus hold the key to detecting associations in the presence of genetic heterogeneity, where the genetic component of disease-risk is determined by multiple rare mutations, each with a low marginal effect on disease-risk (i.e. low population attributable risk; PAR). Recent studies support the hypothesis that multiple rare mutations, each with a low marginal effect, may be a major player in genetic determination of susceptibility for some complex diseases [3]–[13]. Examples of genetically heterogeneous diseases include cystic fibrosis [14],[15], colorectal cancer [16] and probably schizophrenia [13]. Different genetic models may underlie genetic heterogeneity. One possibility is that multiple different variants located across the genome have independent influence on disease risk, such that each variant explains only a small fraction of all affected individuals. Another scenario is that the function of each haplotype of a gene is destroyed if one (or more) lethal mutations occur on the haplotype. In this manner, an individual must have at least one mutation on each of the two haplotypes to be predisposed for the disease (see the Recessive-Set model in Figure 1). In both of these models, the marginal PAR of each mutation may be very low, even when the disease is highly heritable.

Association studies using panels of common SNPs are well suited for identifying variants each with a relatively high PAR, whereas multiple rare variants, each with a small PAR, are difficult to identify using these methods [17]–[24]. In cases where a single (or very few) common variants are expected to be associated with a disease, a variant-by-variant approach using the strongest marginal signal for each tested variant may be beneficial (as discussed in [25] and [26]). On the other hand, when multiple rare mutations are expected to influence disease risk, an obvious approach is to group the variants according to function, such as genes, pathways and ultra conserved regions, and compare the group counts rather than the counts for each variant in the group. The rationale behind this grouping approach is that if many different mutations in a group affect disease risk, it may be beneficial to focus on the group rather than on each variant individually.

The cohort allelic sums test (CAST) is an existing grouping method in which the number of individuals with one or more mutations in a group (e.g. gene) is compared between affected and unaffected individuals [5],[26],[27]. An alternative method using a grouping approach is the Combined Multivariate and Collapsing (CMC) method [26]. In this method all rare variants are collapsed, as in the CAST method, and the collapsed variants are treated as a single common variant which is analysed together with the other common variants using multivariate analysis [26]. In the CMC version used in [26], rare variants are defined as those having a minor allele frequency (MAF) of at most 1%.

In this study, we focus on a scenario in which a group of multiple rare mutations has been identified. In functional regions, one may choose to include only probable disease susceptibility mutations (non-synonymous substitutions, frame shift mutations, etc) in the group of mutations. Using only probable disease susceptibility mutations has the benefit that random variation due to non-associated variants may decrease. In this manner, association studies of groups of rare probable disease-susceptibility variants may be able to identify genetically heterogeneous mutations, and hence complement genome-wide analysis of common SNPs. Grouping of mutations according to functional elements, such as genes, has the added advantage of focusing on causal relations between genes and diseases, rather than just identifying highly associated genomic regions. Furthermore, since many (millions of) mutations are expected to be identified in a resequencing study of thousands of individuals [28], grouping lowers the burden of multiple testing.

We propose a weighted-sum method in which mutations are grouped according to function (e.g. gene), and each individual is scored by a weighted sum of the mutation counts. To test for an excess of mutations in affected individuals, we use permutation of disease status among affected and unaffected individuals. By using permutation, the method adjusts for the weighting of the mutations and the requirement that a mutation must be observed to be included in the study. Note that permutation of disease status results in correct type I error even in the presence of linkage disequilibrium (LD) [29],[30], although relatively low LD is expected between rare variants [26],[31],[32].

The weighted-sum method deviates from the CAST method [5],[27] by weighting the variants differently when determining the genetic load of an individual. By weighting the signals from each mutation, the weighted sum method accentuates mutations that are rare in the unaffected individuals, so that the test is not completely dominated by common mutations. In the CAST method, common variants will have a high impact on the group signal, and if many common mutations are present in a group, almost all individuals will have one or more mutations. To avoid this effect it may be necessary to use a threshold on the mutation-frequencies, as suggested in the CMC method [26]. A drawback of such frequency thresholds is that it can be difficult to select them in a biological meaningful way, and the outcome of the test will depend on the selection of thresholds. In the weighted-sum method we include mutations of all frequencies, but mutations are weighted according to their frequency in the unaffected individuals.

## Methods

### Weighted-Sum Method

The weighted-sum method compares the number of mutations in a group of variants between samples of affected and unaffected unrelated individuals. It is designed to identify an excess of mutations in the affected individuals, compared to the unaffected individuals. Each variant belongs to a group (gene, pathway, ultra conserved area, etc.) and, for a group with *L* variants, the method is comprised of the following steps:

- For each variant *i* ( = 1,…,*L*), we choose which allele of the variant to consider as the mutation (usually this will be the rarer allele, unless other information suggests that the common allele may be implicated in disease susceptibility) and calculate a weight
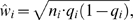
where
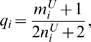
(1)
*m_i_^U^* is the number of mutant alleles observed for variant *i* in the unaffected individuals, *n_i_^U^* is the number of unaffected individuals genotyped for variant *i*, and *n_i_* is the total number of individuals genotyped for variant *i* (affected and unaffected).
  The weight, 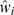, is the estimated standard deviation of the total number of mutations in the sample (including affected and unaffected individuals), under the null hypothesis of no frequency differences between affected and unaffected individuals. It is used to down-weight mutation counts in constructing the weighted-sum score; see (B) and (C) below.
  We estimate *q_i_* according to the mutation-frequency in the unaffected individuals only, rather than the frequency in the combined population of affected and unaffected individuals. We use this approach so that a true signal from an excess of mutations in the affected individuals is not deflated by using the total number of mutations in both affected and unaffected individuals. By using a permutation-based test, we account for using only the unaffected individuals when scaling the mutation frequency, and we are hence able to increase the power of detecting very rare disease-associated mutations. The drawback of this approach is a higher variance of the scaled mutation-frequency, and hence a loss of power when the frequency of the mutation is high. Adding one to the numerator and two to the denominator of the frequency estimate, *q_i_*, avoids zero estimates which would lead to numerical problems in the genetic score used below, and is based on the Bayesian posterior-mean estimate of a binomial proportion when using a uniform prior.
- The genetic score of each individual *j* is calculated as
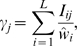
where *I_ij_* is the number of mutations in variant *i* for individual *j*. Under a general genetic model *I_ij_*∈{0,1,2}. However, if a variant (or group) is known to act recessively or dominantly *I_ij_*∈{0,1}, and the components of *m_i_^U^*∈{0,1} accordingly in equation (1); in the recessive case only homozygote mutants are assigned the value 1, and in the dominant case both the heterozygote and homozygote mutants are assigned the value 1.
- All individuals (affected and unaffected together) are ranked according to their genetic scores (γ*_j_*), and the sum of the ranks for affected individuals is calculated as
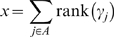
where *A* is the population of affected individuals. Under the null-hypothesis (no disease association) and the assumption that the genotypes of the affected individuals are independent, *x* is a sum of *n^A^* independently and identically distributed (i.i.d.) random variables, and is thus approximately normally distributed according to the central limit theorem. Note that using ranking to determine *x* is equivalent to the procedure in the Wilcoxon test [33].
- The affected/unaffected status is permuted among the individuals, and steps (A)–(C) are repeated *k* times to sample x_1_^*^,…,x_k_^*^ under the null-hypothesis.
- The average (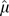) and sample standard deviation (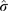) of x_1_^*^,…,x_k_^*^ are calculated and the standardized score-sum is found as
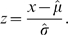
Under the null hypothesis, *z* has an approximately standard normal distribution (see Figure S1 for an example). Thus, a p-value for the association test can be obtained by comparing *z* to the quantiles of the standard normal.

Alternatively a p-value can be found by using a standard permutation test, where the p-value is found by (*k_0_*+1)/(*k*+1), and *k_0_* is the number of the *k* permutations that are at least as extreme as *x*. In such a testing framework, the permuting routine can be stopped if the estimated p-value (and its precision) reaches a certain level; e.g. if the p-value, minus three times the estimated standard deviation of the p-value, is above the significance threshold. Such a permutation strategy may be as fast as the approximation strategy, since fewer than 1000 permutations are needed to reject the hypothesis of association in many cases.

Throughout this paper, the approximation strategy is used because it runs fast for power simulations. Another reason for using the approximation strategy (rather than standard permutation with a stopping rule) is to produce Uniform(0,1) distributed p-values (under the null hypothesis; see Figure S2) for all the tests conducted, which is preferred if further analyses of the p-values are conducted in e.g. a pathway analysis. The standard permutation approach can only produce uniformly distributed p-values under the null hypothesis if no stopping rule is used, which is a computationally expensive approach.

Whether using the approximation or standard permutation strategy, permutation of the case-control labels maintains the LD structure of the genetic data. Thus, the test is valid (i.e. has correct false positive rate) whether or not the variants are in LD.

### Power Simulations

The weighted-sum method is compared to the CAST, CMC, and variant-by-variant methods, which were discussed in the introduction and are described in more detail in Comparison with other Methods. For each set of parameters, 100 datasets are simulated, the four methods are applied, and the proportions of significant outcomes are used as the power estimates. To mimic a genome wide study of about 20,000 fairly independent human genes, we calculate a p-value for each gene, and use a significance threshold of 0.05/20000 = 2.5×10^−6^ in all power simulations.

#### Genetic Models

Four genetic models are investigated (see Figure 1). For the Recessive, Additive and Dominant models the disease-related variants act independently, whereas for the Recessive-Set model the outcome of a mutation at one variant depends on the presence of a mutation at another variant (see Figure 1). We do not sample Dominant-Set or Additive-Set models, since in these models the heterozygote predisposes for disease, and hence they perform like the Dominant and Additive models respectively. We sample the variants independently for simplicity and because rare variants are not expected to be in high LD with the surrounding variants [31],[32].

#### Frequency Spectra

For the Recessive, Additive and Dominant models, we sampled the unaffected population frequency spectrum of the mutations at each variant according to Wright's formula [34],[35]:
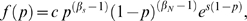
where *f*(*p*) is the probability function of the mutation-probability *p*, *β_S_* is the scaled mutation rate of disease mutations, 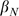 is the scaled back-mutation rate and *s* is the scaled selection rate [32]. The constant *c* normalizes the integral of *f*(*p*) to 1. The frequency spectrum for each variant is sampled with parameters for mildly deleterious mutations, *β_S_* = 0.001, 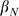 = *β_S_*/3 and *s* = 12, as discussed by [32].

For computational simplicity, under the Recessive-Set model, mutations are drawn with the same probability for each variant in a group. The mutation probability is calculated such that the probability (*p_M_*) that a haplotype contains at least one disease-risk mutation is fixed in unaffected individuals. In concordance with human resequencing studies we use *p_M_* = 10% as baseline [5],[16], but we have investigated other values also.

#### Sampling Individuals

To control the PAR (population attributable risk) of each group, and ensure that all variants have a low effect, we sample each variant in a group using the same marginal PAR (*α*), so that *α* is the group-PAR divided by the number of disease-risk contributing variants (D-variants). Each variant is sampled independently. The mutation probability in unaffected individuals is sampled according to the frequency spectrum described above, and the genotype probabilities in unaffected individuals are calculated assuming Hardy-Weinberg proportions. The odds ratio (*r*) of each genotype is calculated from the genotype probability in the unaffected individuals (*q_U_*) using
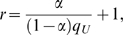
and the genotype probability in the affected individuals (*q_A_*) is calculated as
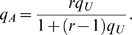
See e.g. Ref. [36]. The population of affected and population of unaffected individuals are sampled using *q_A_* and *q_U_* respectively. We simulate *n^A^* = 1000 affected and *n^U^* = 1000 unaffected individuals unless otherwise stated.

#### Disease-risk contributing variants and disease-risk neutral variants

Because not all probable disease susceptibility mutations (non-synonymous substitutions, frameshift mutations etc.) contribute to disease-risk, we simulate both disease-risk contributing variants (D-variants) and disease-risk neutral variants (N-variants). Under all genetic models, the N-variants are sampled with the same genotype probabilities in affected and unaffected individuals, and the frequency spectrum of mutations follows Wright's formula.

It has been reported that about 70% of all rare missense mutations are deleterious [4], but since not all deleterious mutations necessarily contribute to disease-risk, we simulate 50% D-variants as the baseline, but investigate other levels also (see Results). As discussed in [32], a human gene may contain up to 1000 disease susceptibility variants, whereof only a part are polymorphic in a given sample. Resequencing studies of the coding parts of human genes suggest that 50 disease susceptibility variants is a realistic level [5],[7],[16], and we therefore simulate groups with 50 D-variants and 50 N-variants as the baseline, but investigate other levels also (see Results).

#### Tested Variants

The mutation probabilities (*p*) can be very low for some of the sampled variants. This means that some variants contain no mutations in any of the sampled individuals, and these variants are hence omitted in the tests.

### Encode Data

To evaluate the weighted-sum method on rare variants with the frequency-spectrum of a naturally occurring population, we used resequencing data from the Encode III project (ftp://ftp.hgsc.bcm.tmc.edu/pub/data/Encode). In the Encode III project ten 100 kb Encode regions were resequenced in different human populations, and all substitutions were identified (see http://www.hgsc.bcm.tmc.edu/projects/human/). To mimic a disease-resequencing study, we grouped all exonic variants of each Encode region, and compared the number of rare variants between the two largest populations: the African YRI population (120 individuals; including 60 individuals from HapMap phase I and II) and the Central European CEU population (119 individuals; including 60 individuals from HapMap phase I and II). Only variants that passed the quality control filter for the ENCODE III study were used (see http://www.hgsc.bcm.tmc.edu/projects/human/). The genotype data were downloaded as the ENCODE III draft release I (on August 11th, 2008), and the “Gencode Ref (encodeGencodeGeneKnownMar07)” track in the UCSC Genome Browser [37] was used to define exon positions in each ENCODE region. Exonic variations were reported for only five of the ten ENCODE regions, and hence only these five regions were used.

### Comparison with Other Methods

The CAST method, as described in [27], corresponds to the method used in [5]. In brief, for each group of variants, it compares the number of individuals with one or more mutations between affected and unaffected individuals, using a standard χ^2^ or Fisher exact test. In this study, we use the Fisher exact test throughout to avoid bias due to distributional approximation.

In the variant-by-variant approach the genotype frequencies of each variant are compared using the one-sided Fisher's exact test, and the significance level of the group is found by Dunn-Sidak correction [38] of the smallest p-value in the group. Note that the Dunn-Sidak correction is very similar to the Bonferroni correction, as the Bonferroni correction is an approximation of the Dunn-Sidak correction. Whereas the Bonferroni correction is slightly conservative for independent tests (such as the independent variants in the power simulations), the Dunn-Sidak correction has the benefit of being exact.

The CMC method is implemented according to the description in [26]. In brief, for the CMC method all rare variants are collapsed, as in the CAST method, and the collapsed variants are treated as a single common variant which is analysed together with the other common variants using multivariate analysis [26]. We used the Fisher product method [42],[43] for multivariate analysis, rather than the Hotelling's *T* ^2^ method, because it allows for one-sided testing, and hence allowed a fair comparison for the CMC method. Note that if a two-sided test were used for the CMC method, the power estimates would then have been too low compared to the variant-by-variant and weighted-sum methods.

The weighted-sum method is implemented as described above, using *k* = 1000 permutations in step C. In all power simulations *I_ij_*∈{0,1,2} is used in step B (even when the dataset is simulated under a recessive or dominant model).

## Results

### Proportion of Variants Containing Mutations

The mutation frequencies are sampled according to Wright's formula (see Methods), and hence mutations are very rare for some variants. Using 1000 affected and 1000 unaffected individuals, mutations are on average observed at only 49.4% of the variants (sd: 4.9%). This means that when e.g. 100 variants are sampled, on average 49.4 variants contain at least one mutation, and are hence tested for association. This level is in concordance with the level from human resequencing studies [5],[7],[16].

### Power versus PAR

**Figure 2:**
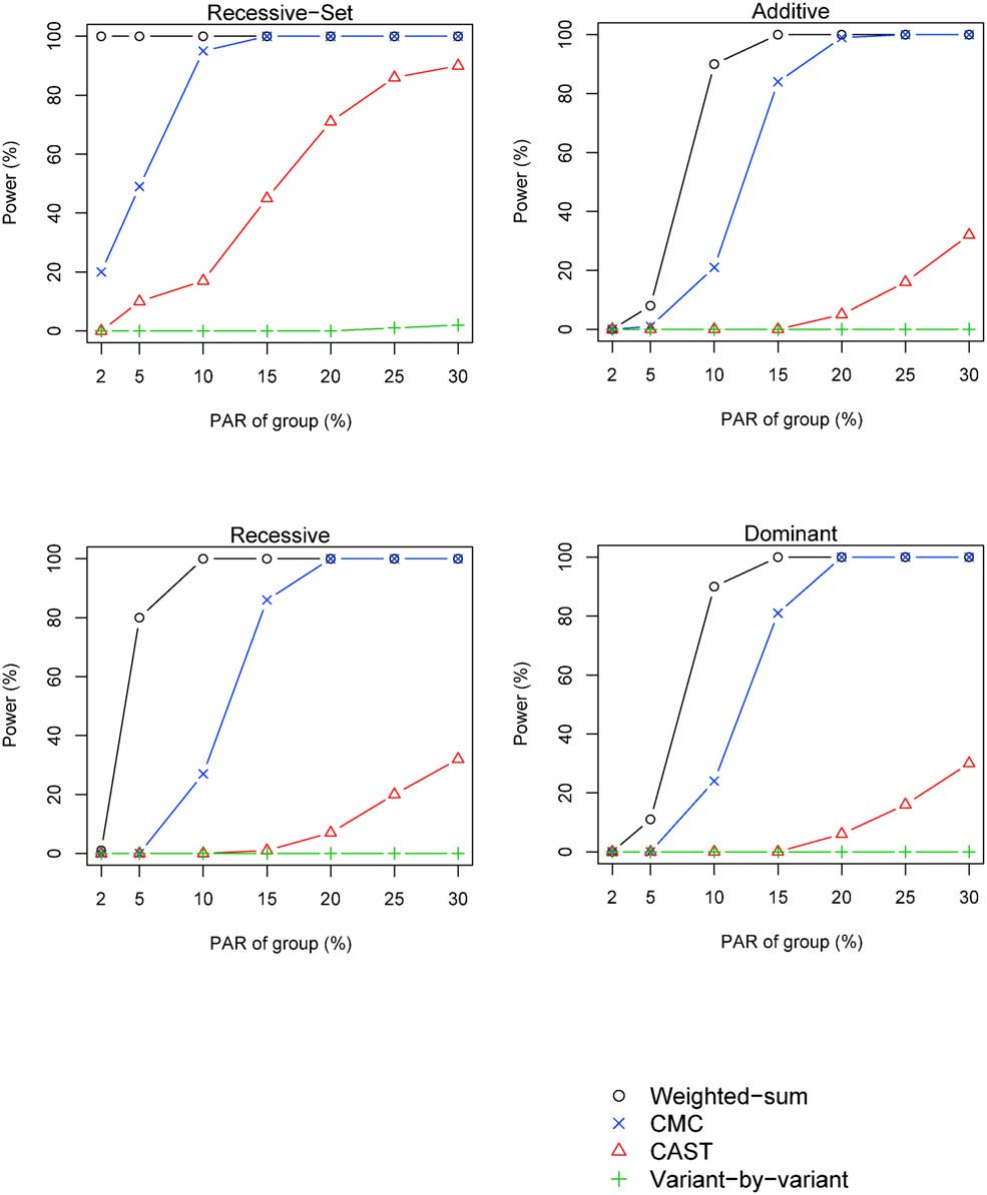
Power versus PAR of group. The power of the investigated methods is shown for different levels of group-PAR. The power simulations were performed using *n^A^* = *n^U^* = 1000 individuals, 50 D-variants, 50 N-variants and *p_M_* = 10%.

Under the baseline parameter settings (see Methods) it is seen that the CMC method, as reported in [26], has better performance than the variant-by-variant and CAST methods, but the weighted sum method has even better performance (Figure 2). The weighted-sum method identifies groups with a PAR of 10%, with at least 80% power, for all genetic models (Figure 2). To investigate whether the weighted-sum method is robust under other model parameters, we fix the group PAR at 10%, and vary the other parameters one by one.

### Power under Varying Model Parameters

**Figure 3:**
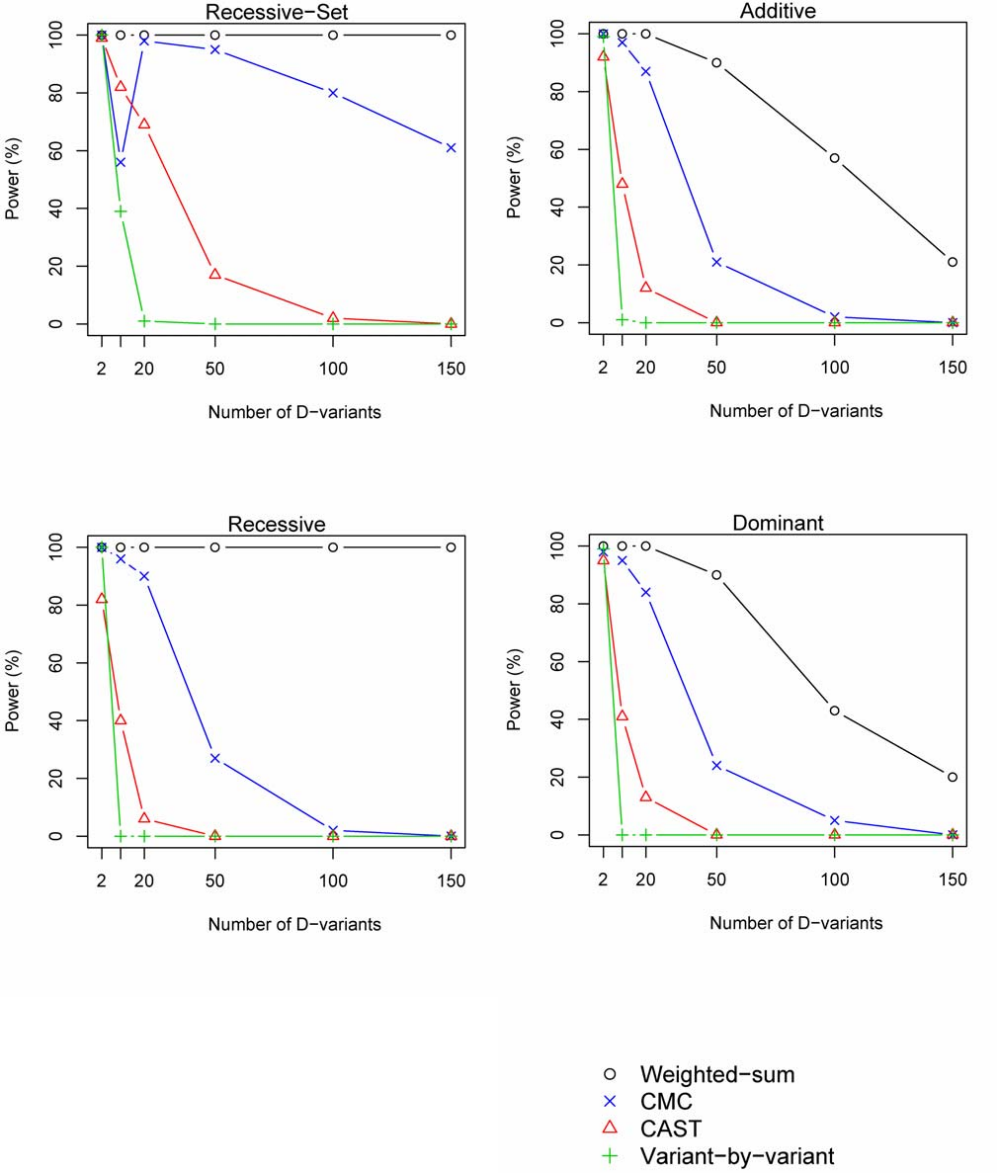
Power versus number of D-variants. The power of the investigated methods is shown for different number of D-variants (disease-risk contributing variants). The power simulations were performed using *n^A^* = *n^U^* = 1000 individuals, 50% D-variants, group PAR of 10% and *p_M_* = 10%. Note that the jump in the power for the CMC method under the Recessive-set model occurs because a low number of variants yields a high allele-frequency of each variant, and the variants are hence not grouped by the CMC method.

The number of variants that contribute to the disease-risk (D-variants) determines the marginal PAR of each variant in the group, such that a low number of D-variants yields a high marginal PAR. Accordingly, all investigated methods perform well when the number of D-variants is low, and hence the marginal PAR is high (Figure 3). When the number of D-variants rises, and hence the marginal PAR of each variant drops, the power to identify a disease-group falls (Figure 3). For the weighted-sum method, the effect of the number of D-variants depends on the genetic model. For the recessive models, it is able to identify even large groups of variants, whereas it is more sensitive to the number of D-variants when the heterozygote contributes to disease-risk (Figure 3).

**Figure 4:**
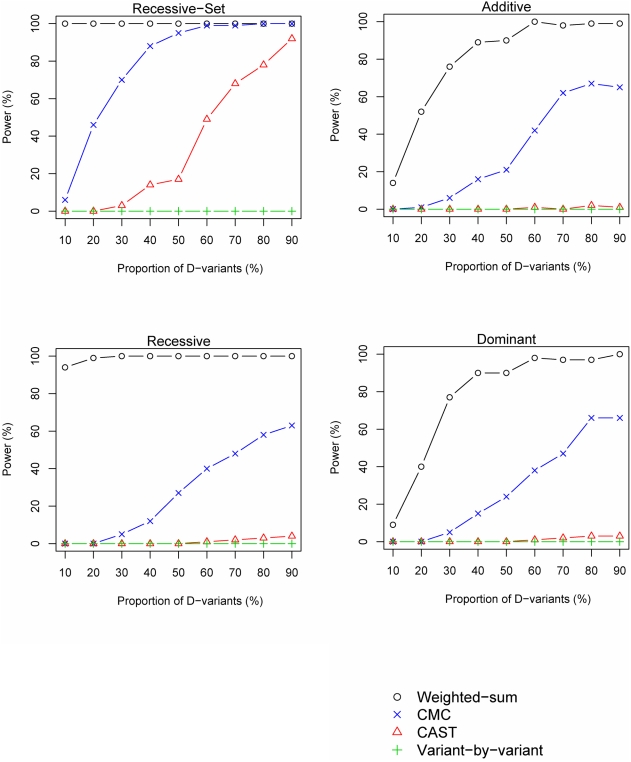
Power versus proportion of D-variants. The power of the investigated methods is shown for different proportions of D-variants (disease-risk contributing variants). The power simulations were performed using *n^A^* = *n^U^* = 1000 individuals, 50 D-variants, group PAR of 10% and *p_M_* = 10%.

The proportion of D-variants likewise influences the power. Under the Recessive-Set model, both the CAST and the CMC methods perform well when a reasonably high proportion of the variants contribute to disease-risk, whereas both the variant-by-variant and the CAST method are unable to identify disease-groups under the other scenarios (Figure 4). On the other hand, the weighted-sum method is generally robust to a low proportion of D-variants in the group, but a higher proportion of D-variants yields higher power (Figure 4).

Note that the probability of mutant-haplotypes (*p_M_*) in unaffected individuals under the Recessive-Set model does not have a large impact on the power (Figure S3).

### Number of Individuals Needed

The number of individuals needed to identify a disease-associated group depends strongly on the underlying genetic scenario. With *n* = *n^A^* = *n^U^* = 1000 individuals, a group with a PAR of 1% can be identified under the Recessive-Set model, while a group with a PAR of 5%–10% can be identified under the other models. A study with *n* = 7000 individuals can identify a group with a PAR of 2% under all genetic models (Table 1; see Tables S1 and S2 for equivalent tables for the CMC and CAST methods).

**Table 1:** Number of individuals needed to identify a disease-associated group.

Recessive-Set							
		*n*					
		500	1000	2000	4000	7000	10000
Group PAR	1	2	**99**	**100**	**100**	**100**	**100**
	2	12	**100**	**100**	**100**	**100**	**100**
	5	18	**100**	**100**	**100**	**100**	**100**
	10	63	**100**	**100**	**100**	**100**	**100**

Recessive							
		*n*					
		500	1000	2000	4000	7000	10000
Group PAR	1	0	0	1	18	66	**95**
	2	0	1	24	**95**	**100**	**100**
	5	0	**80**	**100**	**100**	**100**	**100**
	10	0	**100**	**100**	**100**	**100**	**100**

Additive							
		*n*					
		500	1000	2000	4000	7000	10000
Group PAR	1	0	0	0	2	11	29
	2	0	0	4	29	**88**	**100**
	5	0	8	**84**	**100**	**100**	**100**
	10	1	**90**	**100**	**100**	**100**	**100**

Dominant							
		*n*					
		500	1000	2000	4000	7000	10000
Group PAR	1	0	0	0	2	13	35
	2	0	0	5	33	**93**	**100**
	5	0	11	**85**	**100**	**100**	**100**
	10	0	**90**	**100**	**100**	**100**	**100**

The power (in %) of the weighted-sum method is shown for different numbers of individuals *n* = *n^A^* = *n^U^*, and different levels of group PAR (in %). Combinations with at least 80% power are shown in bold. The power simulations were performed using 50 D-variants, 50 N-variants and *p_M_* = 10%.

### Encode Data

To cover a scenario where the mutation-frequencies are distributed according to a natural existing population, we used resequencing data from 120 individuals from the African YRI population and 119 individuals from the Central European CEU population. In this example, we test for overrepresentation of rare exonic variants in the YRI population compared to the CEU population in each Encode region. Such an overrepresentation is expected since the YRI population generally shows higher diversity than the CEU population [39], and hence more rare variants are expected. Exonic variants are grouped for each ENCODE region, to mimic a disease-resequencing study like the ones reported in human resequencing studies [5],[7],[16]; as a result, 5 groups of 2–72 polymorphic variants are obtained (see Table 2).

**Table 2:** Tests for excess of rare exonic variants in the YRI population compared to the CEU population.

	MAF cut-off				
	1%	2%	3%	4%	5%
**ENm010**					
# variants	42 (30/13)	57 (40/18)	66 (48/20)	69 (51/20)	72 (54/20)
Weighted-sum	2.72×10^−3^	2.22×10^−3^	5.75×10^−6^	5.76×10^−7^	5.44×10^−12^
CMC	2.53×10^−3^	0.01	0.10	0.05	0.01
CAST	2.53×10^−3^	0.01	5.34×10^−4^	4.66×10^−5^	1.21×10^−9^
Variant-by-variant	1.00	1.00	1.00	0.37	0.12
**ENr133**					
# variants	40 (23/20)	43 (26/20)	48 (30/21)	49 (30/22)	51 (32/23)
Weighted-sum	0.41	0.06	3.49×10^−4^	3.22×10^−3^	7.28×10^−4^
CMC	0.51	0.11	0.04	0.04	3.22×10^−3^
CAST	0.51	0.11	1.68×10^−3^	0.03	5.89×10^−3^
Variant-by-variant	1.00	1.00	0.69	0.34	0.04
**ENr232**					
# variants	19 (11/8)	23 (15/9)	28 (19/11)	28 (19/11)	29 (20/11)
Weighted-sum	0.32	0.05	0.02	0.02	4.99×10^−4^
CMC	0.42	0.22	0.20	0.20	6.69×10^−3^
CAST	0.42	0.10	0.07	0.07	4.82×10^−3^
Variant-by-variant	1.00	1.00	0.19	0.19	0.02
**ENr123**					
# variants	4 (3/1)	5 (4/1)	5 (4/1)	5 (4/1)	6 (5/2)
Weighted-sum	0.73	0.21	0.21	0.21	0.97
CMC	0.88	0.35	0.35	0.35	0.57
CAST	0.88	0.35	0.35	0.35	0.98
Variant-by-variant	1.00	1.00	1.00	1.00	0.08
**ENr213**					
# variants	2 (0/2)	2 (0/2)	3 (1/3)	3 (1/3)	4 (2/4)
Weighted-sum	0.93	0.93	0.51	0.51	0.79
CMC	1.00	1.00	0.57	0.57	0.72
CAST	1.00	1.00	0.64	0.64	0.86
Variant-by-variant	1.00	1.00	1.00	1.00	1.00

For each Encode region, we test whether rare exonic variants are overrepresented in the African (YRI) population compared to the central European (CEU) population. To mimic studies of rare variants, five different minor allele frequency (MAF) cut-off values (1%–5%) are used; all variants with a MAF over the cut-off value are omitted in the analysis. For each set of variants, the number of tested variants is reported along with the number of variants that are only polymorphic in the YRI population (the first number in the parenthesis), and the number of variants that are only polymorphic in the CEU population (the second number in the parenthesis). Below the number of variants, p-values from the investigated methods are reported. It is seen that the proposed test yields lower p-values than the alternative tests in nearly all cases where the rare variants are significantly overrepresented in the YRI population. The only exception is for the ENm010 region with MAF cut-off at 1%; in that case, the weighted-sum method yields a slightly higher p-value than the CMC and CAST methods.

As with the simulated data, the weighted-sum method generally shows higher power than the alternative methods to identify an excess of rare variants in the Encode data (Table 2).

Table 2 shows that large groups of variants generally yield lower p-values than small groups. This is expected in the case of heterogeneity, where inclusion of more variants will lead to a stronger combined signal, and hence a lower p-value.

### Computational Speed

In the current un-optimized implementation of the weighted-sum method, a genome wide analysis of 20,000 groups, with 50 polymorphic variants each, using *n^A^* = *n^U^* = 1000 individuals can be completed in approximately 600 CPU hours on a standard stand-alone machine (Intel Pentium Dual 2 GHz, 2GB RAM). When the number of permutations (*k*) is 500 instead of 1000, the results are unaffected (results not shown) but the computing time is halved, however since the test is fast we use *k* = 1000 in this study. Note that the computation time is linear in number of individuals and number of permutations (see Table S3).

## Discussion

In this work, we propose a specialised method to identify multiple rare mutations underlying a genetically heterogeneous disease. Analysis of real data and power simulations show that the proposed weighted-sum method performs very well compared to existing methods. This demonstrates that the use of specialised analytical methods can improve power to identify genetic components of complex (genetically heterogeneous) diseases. On the other hand, it must be kept in mind that the power of such specialisation is at the cost of generality, and therefore the methods must be used in combination with other strategies covering other biological scenarios such as the common variant common disease scenario. It must further be noticed that all methods using the grouping approach (i.e. CMC, CAST and weighted-sum) are sensitive to misclassification of which allele is treated as the mutation (i.e. disease-related allele). If disease-related alleles from some variants are grouped with wild-type alleles from other variants it may hide a true signal. As stated in the Background section, it may be natural to treat e.g. non-synonymous substitutions, frame shift indels and very rare alleles as mutations, but when there is no information to classify the alleles, grouping methods may not be useful. Instead the idea from the CMC method can be used, such that the variants that can be grouped are analysed with a grouping statistic (e.g. the weighted-sum method), and all other variants are analysed variant by variant or by multivariate analysis.

The weighted-sum method is designed for resequencing data, since this technology allows rare mutations to be observed directly. The use of inferred haplotypes from tag SNP studies is a current approach to evaluation of unobserved variants, but this approach fails when the unobserved variants are rare; the tag SNP approach is hence not suited for the scenario of multiple rare disease-mutations [2]. Alternatively, familial linkage studies are a strategy to identify mutations underlying genetically heterogeneous diseases, but when the marginal effect of each mutation is low, it may be difficult to obtain a sufficient number of affected individuals to detect a disease association [40],[41].

The weighted-sum method can be adapted to a wide range of study designs, by e.g. the following: (A) Using the posterior probability of each genotype rather than the most probable genotype. (B) Analysing mutations in conserved areas by weighting each mutation according to the measure of conservation; this is an extension of the conservation base selection criterion from [7]. (C) Analysing continuous traits by testing for correlation between genetic ranks (or scores) and the trait measure. Furthermore, the weighted-sum method can be used for other types of data that can be grouped according to function. Such data include for example methylation measures, where multiple regions/sites can be methylated in promotor regions (i.e. the CpG islands). Note that ranking can be omitted in the test procedure, so the test statistic is the sum of the genetic scores (γ*_i_*) of all affected individuals, rather than the sum of ranks. In the tests performed in this study, the two procedures yield very similar results (results not shown), but we prefer to use the ranking procedure because it is robust to outliers.

The mutation weights (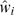) can be chosen in an infinite number of ways. We suggest using the estimated standard deviation of the total number of mutations in the sample (including affected and unaffected individuals), under the null hypothesis of no frequency differences between affected and unaffected individuals. This choice of weight ensures that all variants in a group contribute equally to the weighted sum, under the null hypothesis. The weight of each mutation is determined by its frequency in the population of unaffected individuals only. In this way, a mutation which is common among unaffected individuals has lower weight than a mutation which is rare among the unaffected individuals. If further information about the mutations is available, it may be incorporated in the weights. Such information could include the estimated impact of a mutation or a measure of conservation of the surrounding region (as discussed above).

Analysis of pathways can be done in two different ways. One way is to use the pathway as a group, and run the test on the entire pathway. On the other hand, for large pathways, it may be beneficial to use a method that allows a gene with a strong signal to have a high impact on the combined pathway test-statistic (*T*). If a pathway contains *G* non-overlapping genes, a method to do this is to use the weighted-sum method on each gene, and combine the resulting p-values (*π*_1_,…,*π_G_*) with the Fisher product test statistic
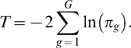
Since *π*_1_,…,*π_G_* are i.i.d. uniformly(0,1) distributed under the null-hypothesis, *T* is χ^2^-distributed with 2*G* degrees of freedom, and can be evaluated accordingly [42],[43]. This method allows for fast analysis of different pathways, using the results from the gene-analysis, and can thereby assist in the functional analysis of a disease association study.

Simulating inheritance of a genetically heterogeneous disease can be performed in different ways. To ensure that all variants have a low effect, we have chosen to simulate all variants within a group with the same PAR. An alternative scenario is to simulate all variants, in a group, with the same relative risk (RR), and let the PAR vary according to the mutation-frequency. Under this scenario, a single, or few, common mutations may carry a large part of the total risk, and this scenario is hence equivalent to a scenario with a single, or few, disease-contributing variants. A few common variants carrying a relatively large risk is exactly the what studies using panels of SNPs are designed for, and our focus has therefore been on scenarios where the disease risk can not be explained by a few variants. Note further that all investigated methods are able to identify cases where a few mutations carry a large part of the total risk (see Figure 3). We have further included the comparison of the Encode populations, to cover a scenario where the mutation-frequencies are distributed according to an actual population.

In summary, we show that the weighted-sum method is powerful for identifying multiple rare mutations underlying genetically heterogeneous diseases. Under some genetic scenarios, 1000 affected and 1000 unaffected individuals are sufficient to identify e.g. a gene with a PAR of only 1%, corresponding to an odds ratio of 1.1. These findings thus demonstrate that resequencing studies have the potential to identify important genetic associations, provided specialised analysis methods are used.

## Supporting Information

Figure S1 Distribution of permuted ranked score sums x_1_^*^,…,x_k_^*^ for ENCODE region ENm010. The distribution of the ranked score sums (x_1_^*^,…,x_k_^*^) from the *k* = 1000 permutations is consistent with normality, as the points follow the line of identity. The data set is an example containing all exonic variants with MAF≤5% from the ENCODE III project, region ENm010 (see Encode Data in Methods for details). The permuted data (x_1_^*^,…,x_k_^*^) show similar Gaussian properties for the other tested scenarios (data not shown).
(.006 MB TIF)

Figure S2 Distribution of p-values under the null hypothesis of no disease association. The distribution of 20,000 p-values under the null hypothesis is consistent with a uniform distribution, as the points follow the line of identity. The simulations were performed using *n^A^* = *n^U^* = 1000 individuals and 100 N-variants.
(.005 MB TIF)

Figure S3 Power versus probability of mutant-haplotypes in the Recessive-Set model. The power of the investigated methods is shown for different levels of probability of mutant-haplotypes (*p_M_*). The power simulations were performed using *n^A^* = *n^U^* = 1000 individuals, 50 D-variants, 50 N-variants and group PAR of 10%.
(0.6 MB TIF)

Table S1 Number of individuals needed to identify a disease-associated group, using the CMC method. The power (in %) of the CMC method is shown for different number of individuals *n* = *n^A^* = *n^U^*, and different levels of group PAR (in %). The power simulations were performed using 50 D-variants, 50 N-variants and *p_M_* = 10%.
(0.02 MB PDF)

Table S2 Number of individuals needed to identify a disease-associated group, using the CAST method. The power (in %) of the CAST method is shown for different number of individuals *n* = *n^A^* = *n^U^*, and different levels of group PAR (in %). The power simulations were performed using 50 D-variants, 50 N-variants and *p_M_* = 10%.
(0.02 MB PDF)

Table S3 Computational Speed. The computation time (in CPU hours) is shown for testing 20,000 groups with 50 polymorphic variants each, using the weighted-sum method. The speed computation is done for different number of individuals (*n* = *n^A^* = *n^U^*), and different number of permutations (*k*). It is seen that the computation time is linear in the number of individuals and in the number of permutations.
(0.01 MB PDF)
