# Supplementary figures and images for: A Groupwise Association Test for Rare Mutations Using a Weighted Sum Statistic
Source: PLoS Genet. 2009 Feb 13;5(2):e1000384. doi: 10.1371/journal.pgen.1000384 (PMC2633048; doi:10.1371/journal.pgen.1000384)

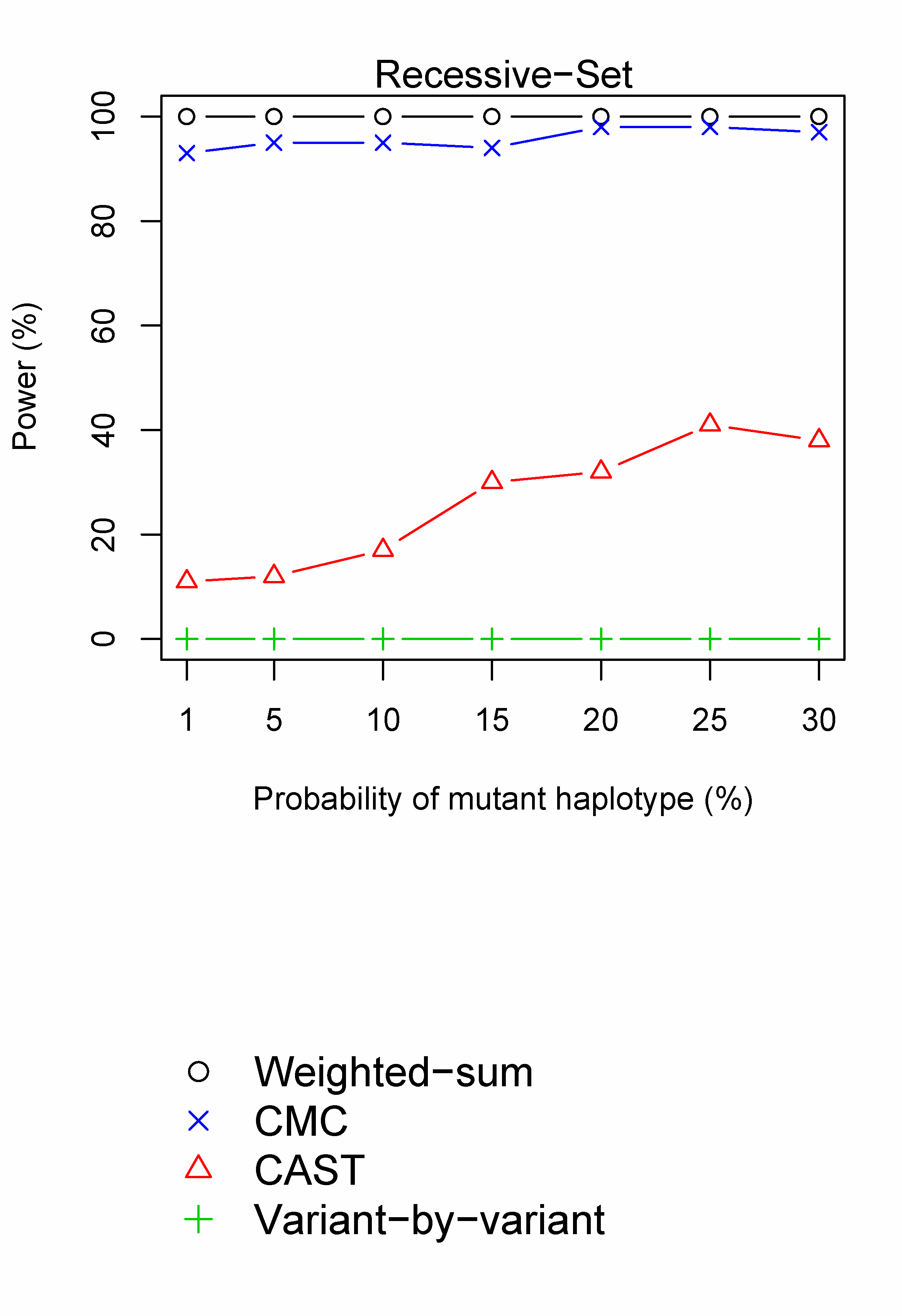

Supplement: Figure S3 — Power versus probability of mutant-haplotypes in the Recessive-Set model. The power of the investigated methods is shown for different levels of probability of mutant-haplotypes (pM). The power simulations were performed using nA = nU = 1000 individuals, 50 D-variants, 50 N-variants and group PAR of 10%. (0.6 MB TIF) [file pgen.1000384.s003.tif]
