# Supplementary material for: A Groupwise Association Test for Rare Mutations Using a Weighted Sum Statistic
Source: PLoS Genet. 2009 Feb 13;5(2):e1000384. doi: 10.1371/journal.pgen.1000384 (PMC2633048; doi:10.1371/journal.pgen.1000384)
Supplement: Table S1 — Number of individuals needed to identify a disease-associated group, using the CMC method. The power (in %) of the CMC method is shown for different number of individuals n = nA = nU, and different levels of group PAR (in %). The power simulations were performed using 50 D-variants, 50 N-variants and pM = 10%. (0.02 MB PDF) [file pgen.1000384.s004.pdf]

A: Recessive-Set

		n					
		500	1000	2000	4000	7000	10000
Group	1	0	13	59	98	100	100
PAR	2	1	16	79	100	100	100
	5	9	57	97	100	100	100
	10	46	96	100	100	100	100

B: Recessive

		n					
		500	1000	2000	4000	7000	10000
Group	1	0	0	0	0	0	0
PAR	2	0	0	0	0	1	2
	5	0	0	5	39	87	96
	10	2	28	86	100	100	100

C: Additive

		n					
		500	1000	2000	4000	7000	10000
Group	1	1	0	0	0	0	0
PAR	2	2	0	0	0	1	1
	5	5	0	1	4	38	75
	10	10	1	22	82	100	100

D: Dominant

		n					
		500	1000	2000	4000	7000	10000
Group	1	0	0	0	0	0	0
PAR	2	0	0	0	0	1	5
	5	0	1	6	33	81	96
	10	1	29	84	100	100	100
