# Supplementary material for: A Groupwise Association Test for Rare Mutations Using a Weighted Sum Statistic
Source: PLoS Genet. 2009 Feb 13;5(2):e1000384. doi: 10.1371/journal.pgen.1000384 (PMC2633048; doi:10.1371/journal.pgen.1000384)
Supplement: Table S2 — Number of individuals needed to identify a disease-associated group, using the CAST method. The power (in %) of the CAST method is shown for different number of individuals n = nA = nU, and different levels of group PAR (in %). The power simulations were performed using 50 D-variants, 50 N-variants and pM = 10%. (0.02 MB PDF) [file pgen.1000384.s005.pdf]

A: Recessive-Set

		n					
		500	1000	2000	4000	7000	10000
Group	1	0	1	4	34	66	80
PAR	2	0	1	10	38	66	86
	5	0	5	31	77	92	95
	10	6	21	56	88	99	100

B: Recessive

		n					
		500	1000	2000	4000	7000	10000
Group	1	0	0	0	0	0	0
PAR	2	0	0	0	0	0	0
	5	0	0	0	0	2	4
	10	0	1	0	11	22	39

C: Additive

		n					
		500	1000	2000	4000	7000	10000
Group	1	0	0	0	0	0	0
PAR	2	0	0	0	0	0	0
	5	0	0	0	0	1	3
	10	0	0	0	6	29	38

D: Dominant

		n					
		500	1000	2000	4000	7000	10000
Group	1	0	0	0	0	0	0
PAR	2	0	0	0	0	0	0
	5	0	0	0	0	3	4
	10	0	0	1	11	27	38
