# Supplementary material for: A Groupwise Association Test for Rare Mutations Using a Weighted Sum Statistic
Source: PLoS Genet. 2009 Feb 13;5(2):e1000384. doi: 10.1371/journal.pgen.1000384 (PMC2633048; doi:10.1371/journal.pgen.1000384)
Supplement: Table S3 — Computational Speed. The computation time (in CPU hours) is shown for testing 20,000 groups with 50 polymorphic variants each, using the weighted-sum method. The speed computation is done for different number of individuals (n = nA = nU), and different number of permutations (k). It is seen that the computation time is linear in the number of individuals and in the number of permutations. (0.01 MB PDF) [file pgen.1000384.s006.pdf]

		n			
		250	500	750	1000
	250	38	75	112	151
k	500	77	150	225	299
	750	116	226	337	447
	1000	154	301	449	596
